# Comparison of different methods for delayed post-mortem diagnosis of falciparum malaria

**DOI:** 10.1186/1475-2875-8-244

**Published:** 2009-10-28

**Authors:** Nicole Berens-Riha, Inga Sinicina, Erna Fleischmann, Thomas Löscher

**Affiliations:** 1 Abteilung fuer Infektions- und Tropenmedizin (AITM), Klinikum der LMU, Ludwig-Maximilians-Universitaet, Leopoldstrasse 5, D-80802 Muenchen, Germany; 2 Institut für Rechtsmedizin, Medizinische Fakultaet der LMU, Ludwig-Maximilians-Universitaet, Nussbaumstrasse 26, D-80336 Muenchen, Germany

## Abstract

**Background:**

Between 10,000 and 12,000 cases of imported malaria are notified in the European Union each year. Despite an excellent health care system, fatalities do occur. In case of advanced autolysis, the post-mortem diagnostic is impaired. Quicker diagnosis could be achieved by using rapid diagnostic malaria tests.

**Methods:**

In order to evaluate different methods for the post-mortem diagnosis of *Plasmodium falciparum *malaria in non-immunes, a study was performed on the basis of forensic autopsies of corpses examined at variable intervals after death in five cases of fatal malaria (with an interval of four hours to five days), and in 20 cases of deaths unrelated to malaria. Detection of parasite DNA by PCR and an immunochromatographic test (ICT) based upon the detection of *P. falciparum *histidine-rich protein 2 (PfHRP2) were compared with the results of microscopic examination of smears from cadaveric blood, histopathological findings, and autopsy results.

**Results:**

In all cases of fatal malaria, post-mortem findings were unsuspicious for the final diagnosis, and autoptic investigations, including histopathology, were only performed because of additional information by police officers and neighbours. Macroscopic findings during autopsy were unspecific. Histopathology confirmed sequestration of erythrocytes and pigment in macrophages in most organs in four patients (not evaluable in one patient due to autolysis). Microscopy of cadaveric blood smears revealed remnants of intraerythrocytic parasites, and was compromised or impossible due to autolysis in two cases. PCR and ICT performed with cadaveric blood were positive in all malaria patients and negative in all controls.

**Conclusion:**

In non-immune fatalities with unclear anamnesis, ICT can be recommended as a sensitive and specific tool for post-mortem malaria diagnosis, which is easier and faster than microscopy, and also applicable when microscopic examination is impossible due to autolysis. PCR is more expensive and time-consuming, but may be used as confirmatory test. In highly endemic areas where asymptomatic parasitaemia is common, confirmation of the diagnosis of malaria as the cause of death has to rely on histopathological findings.

## Background

Malaria is endemic in 109 countries with a disease burden of approximately 250 million clinical cases and 1 million fatalities per year, according to current WHO estimates [1]. At present, between 10,000 and 12,000 cases of imported malaria are notified in the European Union each year, but significant under-reporting is assumed [2].

Despite an excellent health care system with specific and effective therapy options, fatalities do occur in so-called developed countries due to gaps in patient's and physician's knowledge. It is then the pathologist's challenge to discover the truth. In case of advanced autolysis, the histological diagnostic may be hindered or rendered impossible. An uncomplicated procedure like the rapid diagnostic malaria test could facilitate a fast diagnosis.

Rapid diagnostic tests have been developed to enhance malaria diagnostic in areas with limited diagnostic facilities or poor experience. Tests are based on the detection of different malaria proteins, such as *Plasmodium falciparum *histidine-rich-protein 2 (PfHRP2), parasitic lactate dehydrogenase (pLDH), or aldolase. Aldolase is genus-specific, but not species specific. With pLDH a differentiation of all four human pathogen species is possible. PfHRP2 is secreted from infected erythrocytes in peripheral blood and is specific for *Plasmodium falciparum*. ICT PfHRP2 Malaria tests have been evaluated under field conditions [3-6] and compared with PCR [7,8] in endemic regions. Tests were also used in travelers returning from endemic areas under laboratory conditions and as bed-site tests [9-11]. The application of the Malaria ICT for rapid post-mortem diagnosis of falciparum malaria was first published by Sing and colleagues [12]. The reference test in their study was the microscopic diagnosis. The underlying study additionally included a negative control group and molecular investigations for the evaluation of the ICT as post-mortem diagnostic tool for Pf malaria.

## Methods and Patients

The impulse for this study was given by a recent case of fatal malaria in a German traveller returning from Kenya. Massively progressed inner and outer autolysis was diagnosed by autopsy. The corpse had been found about four days after passing away. Pathologically and anatomically no distinct cause of death was traceable. With information obtained from the neighbourhood on recent travel to Africa, malaria was taken into account as differential diagnosis. The parasitological examination consisting of a thin and thick blood smear strengthened the suspicion. Intraerythrocytic bodies consistent with degenerated ring forms of *Plasmodium falciparum *were found. The PCR detecting the 18S rRNA gene of *P. falciparum *[13] confirmed the suspected diagnosis. Also the ICT, specific for *P. falciparum*, was positive. ICT Now (Binax, Inc., Portland, ME) detecting PfHRP2 was applied.

A study to evaluate the ICT for post-mortem diagnosis of malaria was designed. Data of five patients who died of malaria between 2001 and 2008 were therefore collected Microscopic examinations, ICT and PCR tests were performed at AITM. Anamnestic and autopsy findings were known. The control group was recruited at the LMU Institute of Forensic Medicine. From 20 corpses of different age groups found after variable intervals after death testing by ICT, PCR, and blood smear microscopy was performed.

## Results

**Figure 1:**
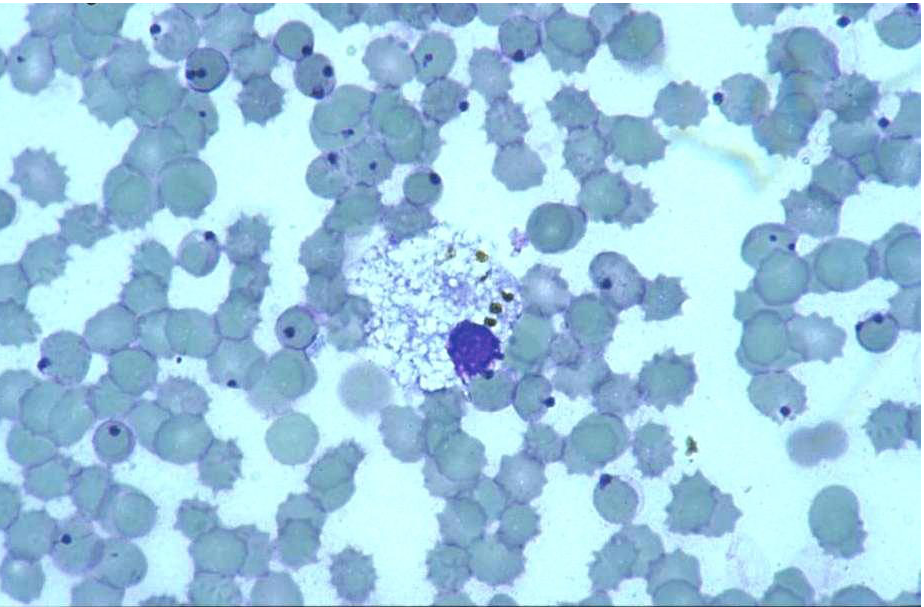
**Blood smear of patient No. 2 with inclusion bodies in preserved erythrocytes suspected as remnants of young trophozoites from *Plasmodium falciparum *and with malaria pigment in an autolytic macrophage**.

The five malaria victims (Tables 1 and 2) were all non-immune travellers returning from tropical East Africa (three females, mean age 58 years). One couple (patient No. 1 and 2) died 2001 of falciparum malaria and was found only several hours later. Remnants of *P. falciparum *trophozoites (ring forms) and immature schizonts could be seen in 50-70% of the erythrocytes as well as intracytoplasmatic pigment in remnants of monocytic blood cells (Figure 1).

**Table 1:** Histological findings at legal autopsy of 5 corpses from malaria patients found at various intervals after their death

	**Patient 1**	**Patient 2**	**Patient 3**	**Patient 4**	**Patient 5**
Brain	Seq.	Seq.	Specimens were destroyed without examination.	Seq. (figure 2)	Seq.
		
Heart	Seq.; MP	Seq.; MP		Seq.; MP	Seq.; MP
		
Lung	Seq.	Seq.		Seq.	Seq.; Inf. Erys
		
Liver	Seq.	Seq.		Fibrosis	Seq.; MP
		
Kidney	Seq.	Seq.		Seq.	Seq.; MP
		
Spleen	n.i.	n.i.		splenectomised	n.i.

Seq.: sequestration of erythrocytes; MP: malaria pigment; n.i.: not investigated due to autolysis

**Table 2:** Malaria cases: comparison of test results

**Malaria Cases**	**Sex(F-M)/Age (y)**	**Interval between death & autopsy**	**Microscopy of blood slides**	**ICT**	**PCR**
Patient 1 (2001)	M/49	4-7 h	Inclusion bodies* in approx. 70% of erythrocytes	Positive	Positive

Patient 2 (2001)	F/53	5-8 h	Inclusion bodies* in approx. 50% of erythrocytes (figure 1)	Positive	Positive

Patient 3 (2004)	F/37	2-3 d	Inclusion bodies* in approx. 65% of erythrocytes	Positive	Positive

Patient 4 (2006)	M/51	4-5 d	Autolytic	Positive	Positive

Patient 5 (2008)	F/63	3-4 d	Partially autolytic, inclusion bodies* in preserved erythrocytes	Positive	Positive

* parasitaemia (% parasitized erythrocytes)

A young female (patient No. 3) with hyperparasitaemia was found dead after 2-3 days. Another patient (patient No. 4) was found several days after his death during a hot summer period. The decay was highly advanced and the blood totally autolytic. No parasites or suggestive remnants were detectable anymore. Patient No. 5 was described above. Only degenerated ring forms could be suspected.

At autopsy marked brain swelling was described in four cases. In patient No. 4, the evaluation was not possible due to advanced decomposition. Petechial haemorrhages in the white matter of the cerebrum, a frequent finding in cases of fatal falciparum malaria, could not be seen macroscopically in any of the patients. The spleen was markedly enlarged in three of five cases (mean weight: 420 g). Patient No. 4 had been splenectomised. The organs of the 63-year-old woman (Patient 5) were decomposed, thus associated with a loss of weight (spleen 250 g).

In all autopsy cases, the information gained from police officers and neighbours but not post-mortem findings were a clue to the tentative diagnosis of malaria. Additional conclusions could sometimes be drawn from the scene of death (e.g., profuse diarrhoea, a thermometer found near the corpse).

**Figure 2:**
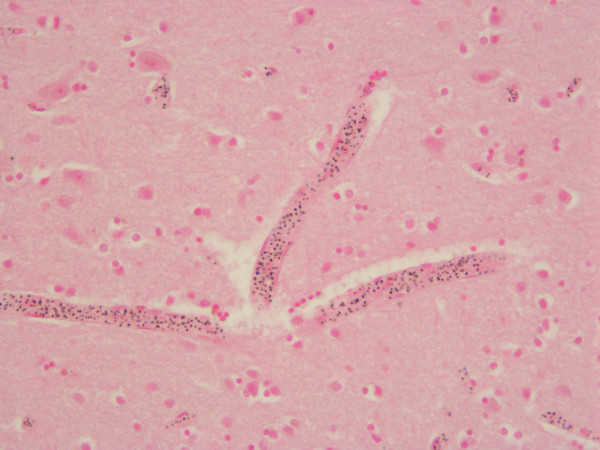
**Brain Histology of patient No. 4 with sequestration of erythrocytes containing *Plasmodium falciarum *schizonts and pigment in post-capillary venules**.

Histopathological examination performed in four cases was compromised by various degrees of autolysis (Table 1). In all patients, sequestration of parasitized erythrocytes in small blood vessels was found in the brain (Figure 2) myocardium, lungs, liver, and to a lesser extent in the kidneys. Scattered petechial haemorrhages in the white matter of the cerebrum were found in three cases. No signs of pneumonia were present in the lungs (Table 1).

ICT and PCR testing was positive for falciparum malaria in all malaria cases (Table 2). The control group consisted of 20 patients who died of trauma, suicide or clearly defined diseases (six females, mean age 64 years). The intervals between death and autopsy varied between five hours to two months. All controls were negative in PCR and ICT. Microscopy showed preserved erythrocytes until day 3. Later on, blood cells were autolytic and no erythrocytes were recognizable. No intraerythrocytic bodies or pigment had been detected (Table 3).

**Table 3:** Control group: comparison of test results

**Control group** **(No. of corpses)**	**Sex (Female-Male)/** **Age (y)**	**Interval between death & autopsy**	**Microscopy of blood slides**	**PCR + ICT**
3	M/28, F/79, F/96	< 24 h	Erythrocytes preserved (all)	Negative

3	M/47, F/56, M/66	24-36 h	Erythrocytes preserved (n = 2), partially autolytic (n = 1),	Negative

3	M/39, M/65, W/69	36-48 h	Partially autolytic (n = 2), autolytic (n = 1)	Negative

5	M/64, M/66, M/68, M/87, F/87	3-5 d	Partially autolytic (n = 1), autolytic (n = 2)	Negative

3	M/61, M/69, F/75	6-9 d	Autolytic (all)	Negative

3	M/38, M/57, M/58	> 9 d*	Autolytic (all)	Negative

## Discussion

The increase of international travel to malaria endemic regions leads to a growing number of malaria cases imported to non-endemic countries, both in non-immune travellers and in immigrants originating from endemic areas who visit friends and relatives in their countries of origin [14]. In non-immunes, falciparum malaria may rapidly result in unlimited parasitaemia, impairment of microcirculation, and tissue damage with multi organ failure. The symptoms in non-immune travellers vary widely and differ from those in immigr ants having some residual immunity. Besides fever, headache (49.7%), myalgia and arthralgia (23.2%), vomiting (11.9%), and diarrhoea (13.9%) were common symptoms of falciparum malaria in non-immunes imported to Europe [15]. Symptoms such as gastrointestinal complaints may be regarded as unspecific or not typical, leading to failures in the diagnostic work-up. However, when appropriate diagnostic procedures (i.e., microscopy and/or rapid tests) are performed timely, most patients can be treated and cured.

Fatalities occur preferably due to lack of awareness in both patients and physicians. The post-mortem diagnosis of malaria may be difficult especially when autolysis and microbiological contamination are advanced such as in cases with an extended interval between death and post-mortem examination. Then, malaria parasites are no longer recognizable during microscopical examination. Malaria pigment can direct to the right diagnosis but can also be misinterpreted or overseen when cell structures are not preserved. Moreover, blood smear microscopy is not a standard examination during autopsy and is only performed in cases of suspected malaria like it happened in our study. Brain swelling and splenomegaly are unspecific findings, and macroscopic signs suggestive of malaria such as petechial hemorrhages in the white matter of the cerebrum have not been present in our case series though histopathology confirmed cerebral haemorrhages in three cases.

The impulse for autoptic examination, including histopathology, was given by the information gained from police officers and neighbours (i.e., recent return from travel to malaria endemic countries) or additional hints.

Histopathology showed typical findings of severe malaria in all four cases examined such as sequestration of parasitized erythrocytes or pigmentation in different organs. However, for pathologists not experienced with malaria cases, misinterpretation of pigment in histological slides as formalin pigment or an artefact seems possible especially when malaria is not suspected or pigment is scarce. Furthermore, histological examinations are not done in every case because they are time-consuming and have to be authorized by the public prosecutor.

Therefore, a simple test for falciparum malaria in cases of an unclear cause of death after travel to endemic areas should be established as standard diagnostic tool that can be performed during autopsy. The sensitivity of the ICT in this study was excellent. In different studies, the sensitivity ranged from 88 - 100% [6,9-11]. This depends upon the density of parasites with a decline of sensitivity (11 - 81%) at low parasitaemia (< 100/μl) [3,16,17]. False negative results in case of *hrp-2 *gene mutations, deletions or antibodies against HRP-2 are discussed [3,16]. They are also possible in patients with high parasitaemia called the prozone phenomenon [9]. The parasitaemia in the study patients was up to 70% and no prozone phenomenon occurred.

Non-falciparum detection is less sensitive [6,18]. Due to lower pathogenicity of other malaria parasites, the post-mortem diagnosis of non-falciparum malaria is negligible.

The specificity of the ICT in this study was high (100%). Different studies showed similar results (80 - 100%). False positive results in rheumatoid factor positive patients have been reported. Cross-reactivity with heterophile antibodies was also discussed [19,20]. Persistent antigen (HRP-2) after treatment is known and must be taken in consideration interpreting a positive test [3,5].

The PCR method is more expensive and time-consuming but can be used for confirmation as it was demonstrated in various epidemiological settings [13,16]. Digestion of the DNA by enzymes due to massive autolysis or bacterial contamination or other disturbing factors has been considered beforehand. But it was not the case in previous studies or the present one. However, only a limited number of cases has been investigated so far. At least there was no false positive case in the control group. If possible, a PCR should be recommended for confirmation as it seems to be also a valid method for post-mortem diagnosis.

In semi-immune populations the validity of microscopy, ICT testing and PCR is limited because asymptomatic infection is common in this population. In this case, only an autopsy with specific histological findings like sequestration of parasitized erythrocytes resulting in multi-organ infarction can reveal the true cause of death. Therefore, ICT testing as a standard tool for post-mortem evaluation in the rare situations where confirmation of the cause of death is required in endemic regions would not be recommended, since asymptomatic parasitaemia can be expected in many patients and also in fatalities due to other reasons.

## Conclusion

ICT diagnostic can be recommended as a sensitive and specific tool for post-mortem malaria diagnosis in non-immune fatalities. The method is easier and faster than microscopy, and also applicable when microscopic examination is impossible due to autolysis. PCR is more expensive and time-consuming but may be used as confirmatory test. In highly endemic areas where asymptomatic parasitaemia is common, the diagnosis of malaria as the cause of death has to rely on histopathological findings.

## Competing interests

The authors declare that they have no competing interests.

## Authors' contributions

EF and NBR performed the laboratory diagnostics. IS examined the corpses and participated in drafting the manuscript. TL designed and supervised the study. NBR coordinated the study and drafted the manuscript. All authors read and approved the final manuscript.
